# Subtle discrimination of fathers in relation to leave-taking: a comparative study of Slovakia and Poland

**DOI:** 10.3389/fsoc.2025.1709287

**Published:** 2026-01-02

**Authors:** Zuzana Dančíková, Magda Muter

**Affiliations:** London School of Economics and Political Science, London, United Kingdom

**Keywords:** parental leaves, fathers, discrimination, paternity leaves, gender-equality, workplace, Poland, Slovakia

## Abstract

The article argues that despite a growing trend of generous leave policies for fathers, leave-related workplace discrimination against them persists, contributing to lower rates of uptake compared to mothers. Using a comparative design, we explore the link between different leave policies for fathers and differences in subtle discrimination of fathers in their workplace when it comes to leave-taking. Drawing on semi-structured individual interviews with fathers and mothers conducted in 2019-20, we compare the cases of Poland and Slovakia, two contexts similar in their inegalitarian gender structures—casting mothers as primary stay-at-home carers of young children and fathers as ideal workers—but different in policies. At the time of our interviews, Poland granted fathers 2 weeks of well-paid, non-transferable paternity leave; Polish fathers could also draw on 32 weeks of well-paid parental leave, which could be divided by parents as they wished. By contrast, Slovak fathers were entitled to 28 weeks of well-paid non-transferable “maternity leave for fathers”. Polish fathers' rate of uptake of paternity leave was the highest, their uptake of parental leave the lowest, with Slovak fathers' uptake of the “maternity leave for fathers” in-between. We find that differences in workplace obstacles linked to the three policies help explain the different rates of uptake. While fathers in Poland rarely reported opposition to taking the short non-transferable paternity leave, Slovak fathers were faced with multiple obstacles to their use of their non-transferable but considerably longer policy, ranging from a lack of established HR processes, through a need to find substitutes for their position to fears of losing their jobs. The obstacles were further exacerbated for Polish parental leave, which parents can divide freely and which is viewed as mothers' entitlement. We conclude that though gendered norms on the division of leaves remain strong in both Poland and Slovakia, casting men as ideal workers and women as primary carers, policy details matter and affect the level of discrimination. Slovak fathers with their non-transferable leave entitlements face comparatively less discrimination than Polish fathers when taking longer leaves. In sum, more egalitarian policy design may help counter subtle workplace discrimination when it comes to fathers' leave-taking.

## Introduction

### Literature review and conceptual framework

#### Fathers' leave uptake limited despite increased desire and enabling policy

Literature has spoken of a rising desire among fathers for a better work-life balance and more engagement with their children (Blithe, 2015; Hobson and Fahlén, 2009). Hand in hand with this desire, the recent decades in the world's rich countries have seen a rise in fathers' involvement in childcare, linked to a cultural shift toward involved fatherhood (Brandth et al., 2022; O'Brien and Wall, 2017), as well as an expansion of dedicated leave policy options for fathers (Brandth and Kvande, 2016; O'Brien and Wall, 2017; Takács, 2020). Despite these developments, compared with mothers, fathers' leave-taking remains limited both in uptake and length across contexts (Haas and Rostgaard, 2011). To account for this discrepancy, Jane Blithe has proposed the metaphor of *glass handcuffs*, which keep men shackled to the labor market, preventing them from greater involvement in the private spere, including in leaves to take care of their children (Blithe, 2015).

#### Explaining fathers' limited leave uptake: linking policy and workplace obstacles

Much research has considered various factors that constrain fathers' leave-taking following the introduction of dedicated policy. Some research has drawn attention to the role of fathers' or their partners' individual attitudes to gender roles or gender equality (Duvander, 2014; Lappegard, 2008). Others have investigated the effect of gendered norms on mothers' and fathers' division of labor (Lott and Klenner, 2018; Marynissen et al., 2019; Mussino et al., 2019), including the ideal parent and ideal worker norms (Lott and Klenner, 2018). The gendered ideal parent norm casts mothers as primary carers and fathers as breadwinners expected to provide for their families economically by continuously working for pay. The ideal worker norm mandates workers are continuously available to their workplace during the day and across the year, assuming no caring obligations, and so aligns with the ideal father norm, but clashes with newly emergent involved fatherhood. Yet other research has pointed to the constraining or enabling role of fathers' workplaces (Browne, 2013; Bygren and Duvander, 2006; Haas and Hwang, 2019; Marynissen et al., 2019; Reimer, 2020).

In response to these various approaches, Zuzana Dančíková has argued for their integration and paying closer attention to their interplay. Building on Barbara Risman's gender structure framework (Risman, 2009, 2017), she posits that men's leave-taking is best explained when conceptualized as shaped by the gender structure (Dančíková, 2025), formed of multiple interconnected gendered factors. Depending on the context, these factors or dimensions of the gender structure may comprise gendered policies that enable fathers' leave-taking, as well as gendered norms or obstacles in fathers' workplaces. In the gender structure framework, a change in one of its dimensions, like the introduction of a new leave policy for fathers, may not only directly affect fathers' leave-taking by enabling or motivating it, but also further shape it indirectly, by shaping other dimensions of the gender structure, by changing norms to support fathers' leave-taking (Bergqvist and Saxonberg, 2017) or by alleviating workplace obstacles. The introduction of dedicated leave policy for fathers may help shift societal and concurrently workplace norms on fathers' leave-taking. At the same time, it may push employers to change their gendered practices to accommodate fathers' breaks from paid work, including introducing HR procedures or trainings for both employees and management. In this paper, we zoom in on the relationship between leave policy for fathers, workplace obstacles and fathers' leave-taking, and explore the ways in which different policies may link to differentiated changes in workplace obstacles for fathers, and consequently, distinct outcomes for fathers' leave-taking.

To operationalize our approach, we draw on the work of Haas and Hwang (2019) who, focusing on the Swedish context, take generous government-mandated policy combined with cultural support for involved fatherhood as a starting point, and explore various mechanisms through which workplaces nevertheless constrain fathers' leave-taking. Haas and Hwang (2019) identify more than two dozen workplace-related obstacles, ranging from a lack of established HR procedures, through a need for fathers to find a solution to filling their position temporarily, to fears about job security. Building on Haas and Hwang's categorization, we explore the ways in which different leave policies, in two similar national contexts, link to differences in the subtle discrimination of fathers in relation to their leave-taking.

#### Workplace obstacles as subtle discrimination of fathers

Much literature has focused on the discrimination of mothers in the workplace (Baszczak et al., 2022; Budig et al., 2012; Cheung et al., 2022; Dawn Metcalfe and Afanassieva, 2005; Glass and Fodor, 2011). Research on motherhood penalties has been prominent (Andersen, 2018; Budig et al., 2012, 2016; Joshi et al., 1999; Pałka, 2024; Weeden et al., 2016)—investigating mothers' lower income compared both to childless or childfree women and men as well as fathers, but ample scholarship is also available on the discrimination of mothers in HR processes (Cheung et al., 2022; Glass and Fodor, 2011). By contrast, fathers have been found to benefit from the fatherhood premium (Weeden et al., 2016; Zajíčková and Zajíček, 2022). The fatherhood premium means increased income or career progression and has been linked to selection effects or their rise in working hours. However, the premium has also been linked to a perception of fathers as more reliable workers due to their familial responsibilities and consequent discrimination of men without children. On the other hand, scholars have also been debating the existence of fatherhood penalties (Weisshaar, 2018; Zajíčková and Zajíček, 2022). In these ongoing debates, researchers have posited that, compared to childless or childfree men, fathers may be discriminated against in terms of loss of income or a lack of career growth after they opt out of the labor market to look after their children. However, we have found little scholarship explicitly focused on the subtle workplace discrimination of fathers when it comes to their intention to take leave in the first place.

We deem that the challenges to fathers' leave-taking that persist in their workplaces despite dedicated leave policy are similar to the subtle discrimination of mothers—the kind of discrimination that persists despite abolishing overt, policy-sanctioned discrimination (Hebl et al., 2020). Consequently, we conceptualize fathers' workplace obstacles as mechanisms of subtle discrimination. This may encompass workplace norms and practices, including insufficient administrative infrastructure and employee awareness of their rights, in so far as they affect fathers differently than men without children or mothers. Subtle discrimination may be intentional or unintentional, but it *is* systematic (Rolin, 2006). Conceptualizing subtle discrimination as workplace obstacles is a move which we find largely absent from the rich literature on fathers' leave-taking, with only limited exceptions (Browne, 2013). Recent books on fathers' leaves eschew explicitly framing the discrepancy between mothers' and fathers' leave-taking in terms of discrimination (Dobrotic et al., 2022; O'Brien and Wall, 2017), as does much research that focuses specifically on workplace obstacles to fathers' leave-taking (Bygren and Duvander, 2006; Haas and Hwang, 2016, 2019; Marynissen et al., 2019). Indeed, literature on fathers' leaves is more likely to invoke workplace discrimination against mothers, linked to their expected or realized breaks from paid work (Gheaus and Robeyns, 2011; Haas and Hwang, 2016, 2019), and present fathers' greater involvement in childcare as a way to address such discrimination against mothers. We maintain that conceptualizing workplace obstacles that fathers face as discrimination rather than mere disadvantage or difficulty provides a more powerful incentive to study and ultimately overcome them. More attention to workplace discrimination of fathers who wish to take leave is called for not only to break fathers' glass handcuffs and facilitate their increased involvement in childcare, but also because fathers' greater uptake of leave can help destigmatize the practice of leave-taking for women and contribute to a more balanced division of leaves and paid and unpaid labor more generally between mothers and fathers (Blithe, 2015).

### Scene-setting: leave policy landscape in Poland and Slovakia

To explore the link between different leave policies for fathers and the subtle discrimination of fathers in the form of persisting workplace obstacles to their leave-taking, we draw on the cases of Poland and Slovakia, two post-communist Central Eastern European (CEE) countries which, compared to Western European countries, have received relatively limited academic attention when it comes to fathers' leave-taking (Dančíková, 2025; Suwada, 2017). These two contexts are well suited to our purpose, as they are characterized by similar gender structures, including inegalitarian norms on parents' division of leaves. At the same time, the respective governments have put in place vastly different policies enabling fathers' leaves, providing a good set-up for a comparative study of the link between policy and workplace obstacles shaping fathers' leave-taking.

Parents' leave-taking in both Poland and Slovakia has been shaped by the norm of threeness (Saxonberg, 2014), mandating that children under the age of three should be at home in the care of family members, typically mothers. The norm has been linked to the bad reputation of nurseries for children under three before the fall of communism, their defunding during the transition to democracy (Saxonberg, 2014; Saxonberg and Szelewa, 2007), and their continued scarcity today. In Poland, fewer than 20% of children aged 0–2 are enrolled in nurseries, in Slovakia fewer than 5% do (OECD Family Database., n.d.). At the same time, the norm of threeness has been linked to maternity, parental and childcare leave policies, which helps explain a stronger norm in Slovakia with its more generous leave policies allowing mothers to stay at home longer, and a weaker, modified one in Poland, where leave policies are less generous.

For decades, policies in both countries facilitated the leave-taking of solely mothers by providing them with leave for a period of up to 3 years of the child's life (Dančíková, 2025; Saxonberg, 2014). After transition to liberal democracy, budgetary cuts affected family policies in the CEE countries (Dawn Metcalfe and Afanassieva, 2005). In 1990s Poland, 16 weeks of maternity leave were in place, extended to 20 weeks in 2000. The maternity leave could be followed by unpaid childcare leave up the child's third year of age, combined with a means-tested benefit (the generosity of the benefit changed with time but since 1990s it remains very limited (Saxonberg and Szelewa, 2007). In Slovakia, 28 weeks of job-protected maternity leave was in place combined with generous, un-taxed maternity benefits. In addition, a 3-year parental leave was available, which could be combined with a universal low, flat-rate benefit now known as parental allowance (Dančíková, 2020).

As a result of the policy set up, leave-taking patterns in both countries were highly gendered. In Poland, mothers often took both maternity and childcare leave and fathers were only allowed to step in and take either if the mother resigned from her right. Statistics concerning childcare leave are not available, but no interviewed Polish father mentioned considering taking such a leave. In Slovakia, mothers typically took maternity leave, followed by parental leave. Similarly to Poland, fathers could step in, if the mother could not take maternity or parental leave. Parental leave was formally extended to fathers in Slovakia in the early 2000s (Zákon, 2002), but it did little to change the existing gendered practice of leave-taking. For instance, in 2020, close to the period when we carried out our interviews, only 3% of all benefits were used by Slovak fathers, while 97% were taken by mothers (Dančíková, 2020).

Overall, for decades fathers both in Poland and Slovakia were perceived only as emergency caregivers, who provided childcare when a mother was unable or unwilling to do so. Dedicated, non-transferable leave for fathers was finally introduced in both countries in 2010, albeit in vastly different ways. Poland granted fathers a week-long paternity leave and extended it to 2 weeks in 2012 (Suwada, 2017). The leave was remunerated at 100% of fathers' previous earnings and to be used within the child's first 2 years of life, including when the mother is on maternity leave (Kurowska et al., 2020). By 2018, close to the time our interviews were conducted, 196 thousand fathers took paternity leave reached (Kurowska et al., 2020), with uptake slightly exceeding 50% (Kelm, 2023).

In Slovakia, 28 weeks of well-paid “maternity benefits”, non-transferable to mothers were made available to fathers in 2011 (Dančíková, 2023). While in Poland, paternity leave was introduced in response to pressures from the EU (Suwada, 2017), the granting of well-paid “maternity benefits” to fathers in Slovakia was the result of a homegrown initiative, and likely unintentional. The benefits were originally meant to be extended to adoptive fathers only, at the same time when the same benefits were also granted to adoptive mothers. However, the legislation was ultimately written in a manner that allowed for use by all fathers with a track record of illness insurance contributions (Dančíková, 2025). Fathers' new maternity benefits could be combined with their pre-existing entitlement to 3 years of parental leave and this combination became commonly known as “maternity leave for fathers”.^1^ The benefit equals 75% of fathers' previous earnings, however, it is not taxed and so amounts to the full income of most fathers, as fewer than 10% of fathers earn more than the generous benefit cap (Dančíková, 2025). The uptake has been much higher than that of the previously available, and much less financially generous parental allowance, reaching almost a quarter of fathers by 2019 (Dančíková, 2023). At the same time, fathers' uptake of this policy was considerably lower than that of Polish paternity leave. This may be because, at 28 weeks, the Slovak “maternity leave for fathers”, is considerably longer than the 2 weeks of Polish paternity leave.

Finally, in 2012, Poland also introduced a parental leave of 32 weeks (Kurowska et al., 2020; Suwada, 2017). At the time of interviews in 2019,^2^ the Polish parental leave was remunerated at 60% of parents' previous income, following maternity leave with 100% income replacement, or—alternatively-−80% for both maternity and parental leaves. Like Slovak parental leave, the policy was seemingly gender-neutral (Plomien, 2019) but the general expectation was that mothers would combine it with maternity leave, and stay at home with the child during the first year of their life. Consequently, despite being ostensibly gender-neutral, the policy is widely seen as extended maternity leave (Suwada, 2017) and overwhelmingly used by mothers. In 2018, 99% of parental leave benefits were taken by mothers (Kurowska et al., 2020). Only 4400 fathers drew on parental leave benefits (Kurowska et al., 2020), a fraction compared to those who took paternity leave.

We suggest that the different leave policies for fathers in the two countries may translate into different workplace obstacles to fathers' leave-taking, which may help explain such different rates of uptake: with significantly longer non-transferable leaves, in the absence of established HR procedures, Slovak fathers may find it more difficult to find a substitute to temporarily take on their responsibilities or may face greater fears about their job security. A similar logic applies to the Polish parental leave.

### Analytical approach, contributions and paper outline

We draw on semi-structured interview data collected for two individual projects. In Poland, Magda Muter collected data on parents' division on paid and unpaid labor in 86 interviews with mothers and fathers. In Slovakia, Zuzana Dančíková focused on fathers' leave-taking and interviewed 38 mothers and fathers. We analyzed the data thematically, focusing on drawing out the similarities and differences in fathers' perception of the mechanisms of subtle discrimination in fathers' workplaces that proved an obstacle to fathers' using the different policies in place in the respective countries.

We find that details of policies regarding paternal leave-taking matter for the workplace resistance that fathers encounter. They influence uptake of leaves, and therefore also largely determine whether various policy aims are achieved. Overall, we find that shorter leaves, clearly attributed to fathers, non-transferable to mothers, meet with less resistance from employers. However, longer leaves may have more transformative potential for gender structure as a whole, and therefore may be necessary to limit subtle discrimination of fathers who take leaves to provide childcare. Our contribution is two-fold. Conceptually, we link two bodies of literature, on workplace discrimination and fathers' leaves, and argue that workplace obstacles to paternal leave-taking amount to discrimination. Empirically, we make a contribution by exploring the workplace obstacles faced by fathers in Poland and Slovakia, two contexts, where fathers' leave-taking has been underexplored.

In the following sections, we introduce our methods and data in greater detail, followed by our analysis, and finally present our conclusions.

## Methods and data

We explore fathers' perception of workplace mechanisms that posed an obstacle to their leave-taking based on interview data collected for two separate projects. In Poland, Magda Muter investigated parents' division of labor following the birth of a child in 2019, including leave-taking. In Slovakia, Zuzana Dančíková explored the effects of a leave policy for fathers in 2019–20, in a combination of in person and online interviews. We both gained ethics approval in line with university guidelines and to ensure open conversations, interviewed the mothers and fathers in our samples separately. We interrogated the validity of our data by conducting all interviews personally, and reflecting on our positionality. We followed our interview guides, to make sure that all respondents spoke to the main areas of our research, including obstacles to leave-taking. Talking to both mothers and fathers helped us triangulate our data, and gave us a more nuanced picture of experiences and decision-making processes about fathers' leave-taking.

Magda Muter's interviews focused on the gendered division of labor, both paid and unpaid, including parents' decision-making regarding said division of labor. The interviews were semi-structured, and the interview guide included a section on leave-taking. Magda Muter recruited participants mainly through her personal acquaintance, social media and snowballing. The data was collected in 86 individual interviews with mothers and fathers. Magda Muter interviewed 43 different-gender couples, in addition, she interviewed one mother and one father whose partners did not take part in the project. Thus, the sample included information on 44 fathers (43 first-hand and one second-hand).

The sample was purpose-designed to comprise of families with at least one young child. Magda Muter mainly interviewed double income couples (at least prior to children) where the youngest child was under seven years old. However, for six couples the youngest child was aged between seven and 16 years old, to diversify the sample according to other criteria. Five such couples lived in a small town/countryside, and one was a less affluent couple from a city. When using snowballing as recruitment procedure, Magda Muter was looking for families with an atypical division of labor, in which men did a lot of housework and childcare. Therefore, the sample includes four fathers who took the unusual path of taking parental leave. However, there were only two couples with a gender-equal division of leaves (with the mother and father taking 6 months each). Most of the fathers interviewed used the paternity leave, and those who did not use it, were mostly not eligible.

Zuzana Dančíková's interviews focused on parents' response to the leave policy for fathers, available since 2011, including parents' decision-making on fathers' leave-taking and factors that shaped their decisions. This focus yielded data on fathers' understanding of obstacles to their leave-taking that they faced in the workplace. Zuzana Dančíková recruited participants through social media, personal acquaintance and snowballing. The data was collected data in 38 semi-structured interviews with mothers and fathers. Zuzana Dančíková interviewed mothers and fathers in 18 different-gender couples, in addition, she interviewed one mother and one father whose partners did not wish to take part in the project. Thus, the sample included information on 20 fathers (19 first-hand and one second-hand). The sample was purpose-designed to comprise both families where fathers did and did not make use of the policy, allowing for insight into both factors complicated fathers' leave-taking and that prevented them from doing so. As a result, the sample included twelve fathers who took leave and eight did not. In addition, only six of the fathers who took leave took complete breaks from paid work, while six carried on with work part-time. They continued with paid work for the previous employer, though two fathers used the time to work on side projects or start a new business.

The samples included fathers working in the public and private sectors, including local and international businesses of various sizes, as well in the third sector (see Table 1 for Poland and Table 2 for Slovakia). The fathers gained various education levels and worked in various positions, ranging from a factory-floor worker to high-ranking managers. However, the samples are skewed toward fathers with higher education, in white-collar jobs, with above-average incomes and located in large cities, including Bratislava and Warsaw. The data was fully transcribed and analyzed using a thematic analysis (Braun and Clarke, 2013). We used NVivo software for coding; the initial code trees were ‘top–down', based on an existing body of knowledge about the gendered division of labor by parents of young children, but we added further codes using a ‘bottom–up' approach (Braun and Clarke, 2006). As mentioned before, our original projects were conducted in continuous conversation with each other. For this paper we additionally worked to ensure thematic reliability by presenting each other with a long list of quotes translated into English specifically on fathers' leave-taking, and then discussing them together at length. Therefore, the analysis of material for this paper was a joint endeavor, which also included a detailed discussion of leave policies and employers' practices in our respective countries.

**Table 1:** Sample of fathers and mothers interviewed in Poland.

**Name**	**Age**	**Education**	**Profession**	**Name**	**Age**	**Education**	**Profession**	**Residence**
M1	31–35	Higher	COO	W1	31–35	Higher	None	Large city
M2	26–30	Higher	Lawyer	W2	36–40	Higher	HR	Large city
M3	36–40	Lower	Clerk	W3	36–40	Higher	Accountant	Large city
M4	26–30	Lower	Teacher	W4	26–30	Lower	Nanny	Large city
M5	36–40	Higher	Designer	W5	36–40	Higher	Designer	Large city
M6	41–45	Higher	Doctor	W6	36–40	Higher	Doctor	Large city
M7	31–35	Higher	Tester (IT)	W7	31–35	Higher	Confectioner	Large city
M8	31–35	Higher	Lawyer	W8	31–35	Higher	Lawyer	Large city
M9	N/A	Higher	IT specialist and manager	Not interviewed	N/A	Higher	IT specialist and manager	Large city
M10	31–35	Higher	Manager IT	W10	31–35	Higher	Designer	Large city
M11	31–35	Lower	Electrician	W11	31–35	Higher	Accountant	Town/Country
M12	36–40	Lower	Driver	W12	36–40	Lower	Saleswoman	Town/Country
M13	36–40	Higher	Copywriter	W13	31–35	Higher	Doctor	Large city
M14	26–30	Lower	Building	W14	< 26	Higher	None	Town/Country
M15	N/A	Higher	Lawyer	W15	31–35	Higher	Kindergarten owner	Large city
M16	31–35	Higher	Environmental specialist	W16	31–35	Higher	Zoo psychologist	Large city
M17	>50	Lower	Technical support consultant	W17	41–45	Higher	Quality control specialist	Large city
M18	36–40	Higher	Project manager	W18	36–40	Higher	Teacher	Large city
M19	31–35	Higher	IT manager	W19	31–35	Lower	Photographer and massage therapist	Large city
M20	36–40	Higher	BHP specialist	W20	36–40	Higher	HR Manager	Large city
M21	36–40	Higher	Data scientist	W21	36–40	Higher	Administrative director	Large city
M22	36–40	Higher	Logistic manager	W22	36–40	Higher	Therapist	Large city
M23	36–40	Higher	Screenwriter	W23	36–40	Higher	Screenwriter	Large city
M24	31–35	Higher	Writer	W24	31–35	Higher	Project manager	Large city
M25	31–35	Lower	Sales representative	W25	31–35	Lower	Office worker	Large city
M26	31–35	Lower	Cook	W26	31–35	Higher	Bank analyst	Large city
M27	31–35	Higher	Optician	W27	26–30	Higher	Chemist	Large city
M28	31–35	Higher	Analyst	W28	26–30	Higher	Analyst	Large city
M29	36–40	Higher	Telecommunications engineer	W29	36–40	Higher	Environmental engineer	Large city
M30	26–30	Higher	Service engineer	W30	26–30	Lower	None	Large city
M31	N/A	Higher	Entrepreneur	W31	31–35	Higher	Teacher	Large city
M32	26–30	Higher	Researcher	W32	31–35	Higher	Computer graphic	Large city
M33	31–35	Higher	Researcher	W33	31–35	Higher	Culture animator	Large city
M34	31–35	Lower	Construction worker	W34	31–35	Higher	Kindergarten worker	Town/Country
M35	26–30	Lower	Vision engineer	W35	26–30	Higher	Pedagogue	Large city
M36	31–35	Higher	Business administrator	W36	31–35	Higher	Project manager	Small city
M37	46–50	Lower	Roofer	W37	41–45	Lower	Cleaner	Town/Country
M38	41–45	Higher	Lacquerer	W38	36–40	Higher	HR Specialist	Town/Country
Not interviewed	N/A	Lower	Upholsterer	W39	36–40	Lower	Tram driver	Town/Country
M40	26–30	Lower	Paver	W40	< 26	Higher	Social worker	Small city
M41	36–40	Lower	Non/Gardener	W41	31–35	Higher	Quality control specialist	Large city
M42	41–45	Lower	Any available	W42	46–50	Lower	Cleaner	Town/Country
M43	46–50	Lower	Digger operator	W43	36–40	Lower	Farmer	Town/Country
M44	26–30	Higher	Policeman	W44	26–30	Higher	Social worker	Town/Country

**Table 2:** Sample of fathers and mothers interviewed in Slovakia.

**Name**	**Age**	**Education**	**Profession**	**Name**	**Age**	**Education**	**Profession**	**Residence**
M1	26–30	Higher	Civil servant	W1	< 26	Lower	Medical student	Large city
M2	26–30	Lower	User experience designer	W2	26–30	Higher	Town planner	Large city
M3	41–45	Higher	Library director	W3	31–35	Higher	Kindergarten teacher	Large city
M4	36–40	Higher	Civil servant	W4	31–35	Higher	Kindergarten teacher	Large city
M5	41–45	Higher	Manager, private corporation	W5	31–35	Higher	Lawyer	Large city
M6	36–40	Higher	Manager, private corporation	W6	36–40	Higher	Manager, private corporation	Large city
M7	31–35	Higher	Schoolteacher	W7	26–30	Higher	Accounting analyst	Large city
M8	36–40	Higher	Manager, private corporation	W8	31–35	Higher	Store owner	Large city
M9	36–40	Higher	Manager, hospitality	W9	36–40	Higher	Structural designer	Town/Country
M10	31–35	Higher	Bank branch manager	W10	31–35	Higher	Civil servant	Large city
M11	31–35	Higher	Contract commercial specialist	W11	26–30	Higher	Training coordinator	Large city
M12	36–40	Lower	Technical foreman	W12	36–40	Higher	Public speaking coach	Large city
M13	36–40	Higher	Schoolteacher	W13	31–35	Higher	Bank branch manager	Town/Country
M14	36–40	Higher	Planning engineer	W14	31–35	Higher	Civil servant	Small city
M15	31–35	Higher	Training coordinator	W15	26–30	Higher	Community coordinator, NGO	Small city
M16	36–40	Lower	Transport foreman	W16	41–45	Lower	Sales assistant	Town/Country
M17	26–30	Lower	Quality technician	Not interviewed	–	Lower	Sales assistant	Town/Country
M18	36–40	Lower	Factory worker	W18	36–40	Lower	Kitchen staff	Town/Country
Not interviewed	31–35	Lower	Construction contractor	W19	< 26	Lower	Sales assistant	Town/Country
M20	41–45	Higher	Manager, private corporation	W20	36–40	Higher	Researcher	Large city

## Findings

To explore the links between policy and workplace obstacles to fathers' leave-taking, we draw on the work of Haas and Hwang (2019), who have identified four themes regarding cultural workplace obstacles to fathers' leave-taking: ‘men's leave-taking is not a strategic company issue', ‘taking leave is not normative', ‘leave-taking should minimally disrupt the workplace', and a ‘job comes first' attitude. They were closely linked to three structural workplace obstacle themes: ‘work practices promoted worker indispensability', ‘insufficient personnel resources', and a ‘lack of infrastructure' facilitating fathers' leave-taking. This framework allows us to consider different kinds of obstacles to leave-taking that fathers in Poland and Slovakia have faced in the workplace. Following Haas and Hwang's framework, we present evidence that fathers are faced with the following obstacles: their leave-taking is not considered a norm, nor a strategic issue for employers; if taking leave, they are expected to put steps in place to avoid causing disruption; and they are under pressure to demonstrate that the job comes first even when taking leave. At the same time, we assess the link between the obstacles encountered and policies in place in the two contexts.

### Men's leave-taking is neither a norm, nor a strategic issue for employers

Haas and Hwang (2019) note that workplace culture in Sweden does not consider men's leave-taking as a ‘strategic company issue', and fathers taking longer leaves is still not a social norm, despite a long history of Swedish state policies supporting more involved fatherhood. Therefore, it is not surprising that in both Poland and Slovakia, where state policies have not been explicitly designed to encourage gender equality, fathers taking long full-time leaves to take care of their children is still not a normative expectation, unlike in the case of mothers.

However, fathers' leave-taking has been increasing, shaped by specific policies available to them. The greatest uptick in leave-taking occurred in Poland, with shorter, well-paid leaves, directly assigned to fathers, rather than couples. In line with the consensus in literature on the effectiveness of well-paid non-transferable leaves for fathers (Dearing, 2016; Haas and Rostgaard, 2011; Moss et al., 2019), when taking leave does not significantly affect the total family income, and the leave cannot be transferred to the mother, fathers are not only less likely to opt out, they also start treating the leave as their right, rather than a special privilege which requires additional justification.

As mentioned in the introduction, there is a striking difference in uptake between Polish paternity leave and parental leave. The paternity leave is short, offers 100% salary replacement, cannot be shared with the mother, and does not require for the father to be on leave alone. On the contrary, the expectation is that most fathers take it directly after the birth of their child, to bond with them and to help the mother. After a decade of this policy, it became “natural” to some Polish fathers to take this leave, and prioritize their family for this short amount of time at the expense of their workplace:

**PL M08** It was natural that the father disappears for 2 weeks just after the birth of a child. And there were no problems with it [in my workplace].

The practice is quite widespread, so that even workers who are officially self-employed, but in reality working as employees of a particular company, a practice common both in Poland and Slovakia due to a lower tax burden on self-employment, are often offered to spend 2 weeks at home following the birth of their child.

**PL M21** The CEO, he had three kids, so it was actually alright from this perspective. Despite being officially self-employed, I got the 2 weeks of paternity leave.

However, even taking the brief paternity leave is not quite the workplace norm yet—demonstrated by the fact that who makes decisions about fathers' leaves still matters. The size of the workplace emerged as a potential mediating factor for the relationship between policy and fathers' workplace discrimination. In small workplaces without an HR department, the personal values of leadership remain crucial. Some Polish respondents reported that, in such unfriendly environments, taking paternity leave remains a challenge, as in the case of the construction worker below, recounted by his wife:

**PL W43** My husband had a problem with his employer, even with taking the paternity leave. In the end he stayed at home.

Though eventually, this father managed to make use of his paternity leave, the lack of normativity is an obstacle that might deter others.

This lack of normativity came across more strongly in Slovakia in relation to the similarly highly remunerated and non-transferable, but considerably longer “maternity leave for fathers”. Whereas, the Polish paternity leave grants fathers 2 weeks off work, the Slovak policy entitles men to 28 weeks of well-paid leave. The length of leave makes taking it much less “natural” amongst Slovaks, including in workplaces, as recounted by the Slovak mother, whose colleagues were ‘astonished' to hear about a man exercising his right to take leave:

**SL W012** I remember mentioning this to friends or colleagues and everybody was like astonished, what? He's going on what? (…) Friends, colleagues, people, which was more surprising, it was people very much around my age, or few years later and they were still like why, but a father is not able to care for a child so small that's sort of the mother's job and I'm like, yeah, not in the twenty-first century.

Though both the Polish and Slovak fathers in the cases above ended up making use of their paternity leave and “maternity leave for fathers”, the non-normativity of fathers' leave-taking remained a greater obstacle for Polish fathers contemplating making use of the parental leave policy. The 32-week parental leave may be well-remunerated (at 80% replacement level, if the mother decides on the 80% replacement rate during maternity leave, too, instead of 100%). Still, for parents with significant differences in salaries, the difference in missing 20% of income may be a factor in decision-making regarding the division of leave. Unlike paternity leave, in 2019, no part of parental leave was reserved for fathers: mothers and fathers were free to share it as they wished. In the absence of a non-transferable daddy quota, it was considered “natural” for mothers to make use of the policy and stay at home with their child. Among Polish respondents there were instances when parents wanted the father to take part of the parental leave but decided against it due to the total novelty of such behavior in the workplace, and expected problems and detrimental effects on the fathers' future career. One Polish mother recounted:

**PL W11** We considered [sharing parental leave], meaning that we thought it would be great. My partner really wanted to take it, but he knew that there would be a huge problem with his employer. There was no situation like that in the company before.

Overall, far from fathers' leave-taking being a strategic issue for employers, like in the Swedish case reported by Haas and Hwang (2019), Polish and Slovak employers often did not expect fathers to take longer leave. However, the differences in policy design seemed to translate into different normativity of fathers' uptake: the Polish brief, well-paid, and non-transferable paternity leave was the most normative and the long-but-transferable parental leave the least, with the similarly long, but both well-paid and non-transferable Slovak “maternity leave for fathers” in the middle.

### Minimal disruption

If fathers decided to take longer leaves, they often experienced administrative and cultural obstacles. Employers were often unprepared, and there was a general expectation that the leave should be as non-disruptive for the employers as possible, which is consistent with the findings of Haas and Hwang (2019). Fathers had to negotiate their time away from paid work for family care, and they were at times expected to solve the problem of managing their workload when they were absent, especially when they themselves held higher positions: job hierarchy emerged as another mediating factor for the relationship between policy and discrimination This was particularly true for longer leaves but occurred with the more normative, shorter leaves, too.

**PL M09** To get 2 weeks of leave to take care of my wife, I had to bring out the big guns. Everyone was involved: my manager, director, Polish general director, the Eastern Europe regional director. It didn't matter to me. I let them know 6 weeks in advance that more or less at that time my wife is going to give birth and that if there is no available replacement in Poland, they can call someone from [other country]. I will train them, check everything, no problem. (...) It was not pleasant and rather tense as always in similar situations but in the end, I had no problems. Few weeks later, I actually participated in a recruitment process for a higher position and I got the job, so the company is not vindicative. At least that… Yeah. We also need to understand the employer's perspective.

The wording used by the Polish father above, who planned 2 weeks of leave to take care of his wife, who was having a caesarean section, shows how the subtle discrimination embedded in the workplace culture is experienced. There is no direct ‘no' from supervisors, but a general tension, the feeling that what you are asking is a favor, rather than your right. The respondent underscored that the company not only did not fire him, but considered, and chose him for a higher position. He finished his story by stressing the need to see the employer's point of view, which shows that having a personal life and family responsibilities is considered a burden for employers, and that this view is accepted by the society, even by employees themselves. It is also important to acknowledge that this father had a very strong negotiating position, as his skills were rare and highly valued. Someone with less confidence or less bargaining power could end up not taking the leave if faced with this kind of tension.

The situation above was atypical for Polish fathers taking 2 weeks of leave after the birth of a child. Most employers did not have such strong reactions to shorter leaves. In this case, the company did not have any infrastructure to support fathers' leave-taking, especially on managerial positions; the respondent was considered indispensable.

In the case of the Slovak “maternity leave for fathers”, similar problems were much more common. The policy granted fathers well-paid, non-transferable benefits for a longer period, yet still left them to face considerable workplace obstacles, which could mirror the length of leave in their extent. One Slovak father recounted the enormous planning and preparation required in advance of his leave, which lasted almost a year, as he tacked on a half year of parental leave on top of his “maternity leave for fathers”

**SL M06** The key success factor was that I started planning it 1 year in advance. With my boss and with my coworkers, and I also prepared everyone gradually, so they can work together well. That was really important.

For this father, the length of required preparation corresponded to the length of his leave. As another father shared, this imperative to ‘prepare everyone' was not an official obligation. However, failing to do so could lead to ‘burning bridges' in the workplace, and fathers usually wanted to continue their careers with their employers after returning to work. As a result, Slovak fathers made efforts to minimize the fallout for their employers:

**SL M03** We had to find a replacement for my position, which proved to be more difficult than we had thought, so the whole process was not without problems. And I could have burned those bridges and said you take care of it, but I had a feeling of responsibility towards my employer, because I have to say my boss met me halfway (...) they didn't make things difficult for me, but a relatively difficult situation occurred, as we couldn't find anything for my position for a long time.

In this case, the father commented that the same problems could occur if he were a mother. This shows that subtle workplace discrimination affects parents, irrespective of gender. At other times, the discrimination of fathers clearly differed from that of mothers. Another Slovak father, when planning to take leave with his older child, felt he could not exercise his right fully to avoid causing disruption:

**SL M02** The first time I asked for three months, I thought more would not be right in relation to my employer (...) The six months would have been a bit brazen, ok, the law allows it, but realistically, they would have had to find someone to do the job instead of me.

His wife, albeit working for a different employer, was faced with no such obstacles and took more than 2 years of leave with the same child. Promisingly, the father took the full 28 weeks or 6 months with his second child, suggesting workplace obstacles may diminish as fathers' leave-taking become more normative. The culture of the workplace may change after the first leave taken by a father with no negative consequences. This is especially true, if this man has a strong position in the company, as in the case of the Polish man described below:

**PL M24** About a month after I returned to work, a colleague who was in a similar situation approached me and said he'd been thinking about taking a longer leave – in the end, he took six months – he did not even ask how the company treats parental leaves for fathers. He just asked what the paperwork was like. I told him truthfully that it wasn't a problem, and he did the same. And I don't know. He might have come to that conclusion without my example, but I think it helped him and gave him a certain confidence.

The need for a role model in one's workplace seems more important for Polish fathers, as the vast majority of Polish respondents never seriously considered the possibility of a father taking a longer leave. Typically, both parents anticipated that the mother will combine maternity and parental leaves and stay at home for the first year of the life of their child, and there was no discussion about leave-sharing.

In sum, both Slovak and Polish fathers who decided to take leaves adjusted the length of leave to avoid disruption and perceived the need to participate in preparing the workplace for their temporary disappearance. Our evidence suggests that the work required to minimize disruption may correspond to the length of absence from the workplace—with more labor required for the longer Slovak “maternity leaves for fathers” compared to the shorter Polish paternity leaves. These experiences speak to a lack of a pre-designed system of replacement, and the need for solutions to be prepared on case-by-case basis.

### Job comes first

Fathers' taking longer leaves was complicated not only by the imperative to minimize disruption for the workplace, but also with the expectation that that ‘job comes first'. Haas and Hwang (2019) identified various ways in which this expectation manifested, including loyalty toward coworkers and thinking about their workload, which is expected to rise due to a father taking leave. In our interviews, we have identified similar themes, with a greater focus on clients and employers, rather than coworkers. This Slovak father recounted how he continued working part-time for his employer once his leave officially started:

**SL M07** In short, I stayed at work for a while, to finalize those relationship things in one job, so my boss would not have to look for a replacement, because activities had been running that had to be finished. And I felt a responsibility to my employer and to clients, for finishing these activities, so they wouldn't stop half-way.

Relatedly, Haas and Hwang (2019) wrote about fear of negative career consequences of leave-taking should fathers not demonstrate that they are putting the job first—including fear of losing one's job altogether. Indeed, some fathers—both in Poland and in Slovakia—were worried about their professional futures, despite the legal protection. For one Slovak father, the fear of losing his job contributed to his decision to not take leave:

**SL M16** The law does say that the employer would have to give the position back to me, but I'm not sure it would really be like that.

Another Slovak father claimed that when he announced he wanted to take leave, it served as an impetus to fire him under a different pretext. Similarly, one of the Polish fathers who prolonged his paternity leave, believed that this was the reason he lost his job:

**PL M14** I used up all leaves, and a sick leave, because my son got sick, too. So, this extended from two weeks [of paternity leave], to over a month and a half (...). And right after returning, I was informed that there was no job for me.

Another Polish father described a company policy of financial incentives, connected strictly to the number of days worked. This was a strong deterrent preventing him from taking any leaves connected to children:

**PL M30** I work in production. (...) In my employer's mind every day when the employee is not at work equals a loss of profit (…). Although I have a full-time contract, the contract is structured in such a way that there is a discretionary bonus added to my salary, and the boss decides what to do. (...) In practice, the bonus is calculated only from the days I'm at work, so let's say that there are 20 working days in a month. I was at work for 15 days, took five days off for my child, then I only get the bonus for 15 days, and the bonus value is also determined by the boss, so yeah.

In sum, men are still faced with the expectation that they their job first, even when they become fathers and have more family obligations. This is a broader concept than minimizing disruption during leave-taking. However, the examples above show that fathers taking longer leave with a child is still uncommon, and therefore seriously disruptive of the image of ‘ideal worker'. This is an especially risky situation for fathers without a strong position in the labor market, whose work may be relatively easily replaced.

## Conclusion

Compared with mothers, fathers' leave-taking has remained limited across the rich world (Haas and Rostgaard, 2011) despite a rise in involved fatherhood that has emerged hand in hand with a proliferation of dedicated leave policies for fathers. In this paper, we zoom in on the relationship between leave policy for fathers, workplaces and fathers' leave-taking, framing persisting workplace obstacles as subtle discrimination rather than mere disadvantage or difficulty. We argue that this framing provides a more powerful incentive to study and ultimately overcome them. The concept of discrimination is commonly used in research of mothers' experiences in the labor market. As fathers increasingly gain similar caregiving rights, with states supporting their role not only as employees, but also parents, fathers face similar challenges to mothers when exercising these rights. It follows that these challenges deserve similar attention, for the sake of fathers as caregivers, but also for mothers to be able to achieve equality as workers. In response, we explore the ways in which different policies in two similar contexts, Poland and Slovakia, translate into different workplace obstacles for fathers wishing to take leave to look after their children. Our contribution is two-fold: first, we contribute the conceptual innovation of framing persisting workplace obstacles as subtle discrimination, and second, we provide new empirical insights on the Central and Eastern European context that remains underrepresented in this field.

We find that details of policies matter for the workplace obstacles that fathers encounter, and they can have significant implications for their uptake of leaves, and therefore for achieving the various policy aims associated with dedicated leaves for fathers, from giving more choice to fathers regarding their engagement in childcare and ensuring fathers' rights (Ellingster, 2012) and children's rights (Brandth and Kvande, 2009), to more gender-equality in the household and in labor market (Haas and Rostgaard, 2011). As highlighted in the introduction, there is a vast international literature regarding discrimination of mothers in the labor market, and some regarding discrimination of fathers. In the CEE context, limited research exists regarding child penalties (Pałka, 2024), mothers' discrimination in the labor market (Baszczak et al., 2022; Ciaputa, 2016; Dawn Metcalfe and Afanassieva, 2005; Mikołajczyk and Stankowska, 2021; Włodarczyk, 2022), and the unequal position of fathers (Hobson and Fahlén, 2009; Włodarczyk, 2022). However, this literature is mainly published in national languages, and there is no special focus on discrimination of fathers in the labor market. Our paper shows how fathers' leave-taking policies shape choices of parents, and limit subtle discrimination of involved fathers on the labor market.

The paternity leave in Poland has had the largest uptake, which is connected to its key characteristics: 100% income replacement, limited length (2 weeks), exclusive and independent right of the father, which means it cannot be transferred to the mother, and finally it may be taken at the same time as maternity leave. Therefore, the father does not have to become a primary carer, nor does he lose his capacity as economic provider. As such, the paternity leave in Poland does provide fathers and children with bonding time but does not undermine the existing unequal gender division of labor. It also causes only minimal disruption to employers, as similar time out of work is often associated with standard vacation time or sick days. Therefore, it only partially challenges the expectation that the ‘job comes first' and is relatively infrequently met with adverse employer reactions.

The “maternity leave for fathers” in Slovakia is also an exclusive and independent right of fathers and has a high replacement rate (dependent on income bracket, but for the vast majority of fathers a 100% income replacement). However, at 28 weeks, the leave is long and although it may be taken together with mothers on parental leave, both parents cannot draw on benefits at the same time. This means that if families are to avoid a loss of income over this relatively longer periods, mothers usually have to return to employment. Therefore, the “maternity leave for fathers” in Slovakia does considerably challenge the existing gender structure, including the workplace norm of the “ideal worker” and the associated workplace culture. This leave length, combined with the non-transferability to mothers are reasons why the “maternity leave for fathers” in Slovakia meets with more resistance from employers than paternity leave in Poland, despite similar gender structures.

Finally, the parental leave in Poland, also rather generous both in terms of length and replacement rate, does not challenge the existing gender structure significantly. Its uptake is highly gendered, with mothers using it overwhelmingly. Unlike the Polish paternity leave and the Slovak “maternity leave for fathers”, the Polish parental leave does not reserve time specifically for fathers. Although seemingly gender neutral (Plomien, 2019), it is implemented within a wider gender structure and confronted with the norm of the mother as main caregiver and the “ideal worker” norm in the labor market. Therefore, it results in very gendered uptake, with mothers almost always taking all available leave, which means it does not undermine existing gendered norms. Most Polish fathers do not experience the parental leave as their right. One of our respondents jokingly “threatened” his employer by saying ‘calm down or I will take a month of parental leave', showing how alien fathers' leave-taking beyond the 2 weeks of paternity leave may still be for employers and fathers alike. Taking parental leave is so strongly against existing norms and patterns of behavior, that some respondents in our sample reflected on being the first father ever in their workplace claiming the leave, even when working for large employers.

Overall, our research has policy implications, as leave policies help set limits to the discrimination of fathers in the labor market. Our main findings are largely consistent with the existing body of knowledge regarding characteristics of effective parental leave for fathers. Firstly, our findings affirm that the leave is more effective in supporting fathers' leave-taking when it is an exclusive and independent right of fathers, rather than a family-based solution (Koslowski and O'Brien, 2022; Kvande, 2022). This is an important policy characteristic which allows for a change in existing patterns of behaviors and, at least to some extent, in existing gender norms on the division of labor. Secondly, our findings also suggest that such non-transferable leave should be highly paid, as this allows fathers to focus on providing care, without undermining their breadwinning capacity. This is crucial in the context of CEE countries where overworking is common (Han et al., 2020), with the work hours of Slovak fathers working high full-time increasing (Aldrich et al., 2016) and with Polish fathers reporting ‘an enormous economic pressure in connection to having children' (Suwada, 2021, p. 55). Thirdly, leave assigned directly to fathers should be of significant length (Malamitsi-Puchner et al., 2023), which will provide an incentive for employers to plan for such fathers' absences, similarly to how they plan for mothers' absences. In turn, lengthy absences should allow fathers to become competent carers, especially when on leave alone (O'Brien and Wall, 2017), rather than together with mothers.

However, to change fathers' leave-taking patterns, more than the right policy mix is needed. Policy implementation also matters. In both Slovakia and Poland, leave policies for fathers were implemented without a dedicated communication strategy (Dančíková, 2023; Plomien, 2019). The respective governments did not actively promote involved fatherhood, nor gender-equality. They have offered a formal choice to parents, but stopped short of challenging existing gendered norms and narratives regarding parenthood. There was also no government support for employers to encourage them to facilitate leave-taking by men. Hence, our recommendation goes beyond long, well-paid and non-transferable leave to include clear communication about the aims of such policy, and—at the very minimum—information materials and trainings for employers and their HR staff.

The rollout of leave policies for fathers may be gradual, and the Polish paternity leave with its high and increasing uptake may be treated as an example of an effective initial step. Such a step should be followed by granting a daddy quota—a part of parental leave—to fathers, not concurrent with paid parental leave for mothers, to encourage fathers to become primary carers. In our view, both parents would ideally have equal individual non-transferable entitlements, which cannot be shared by both parents.

There are two main limitations to our research. Firstly, our samples were skewed toward more educated, relatively affluent residents of Polish and Slovak cities. Secondly, the datasets were obtained from separate projects. Our individual research projects were aligned in many ways, as they not only share similar research interests, and similar context of the fellow CEE countries, but were also conducted in continuous conversation with each other. Still, the projects were focused on the gender division of labor in Poland and fathers' leave-taking in Slovakia, respectively. Hence, while our interview guides did not allow for direct comparison, our broadly collected data allows us to think about workplace obstacles to fathers' leave-taking within the wider framework of the gender structure. However, it also sets limitations to the paper, as workplaces and workplace discrimination were not the original and sole focus of our interviews. With tailored interviews, we could have delved deeper into the variety of workplaces obstacles to fathers' leave-taking. Relatedly, the broader focus of our original projects was also reflected in our recruitment strategies. For instance, we did not strategically recruit fathers from different kinds of workplaces, based on size or sector, and could not meaningfully compare workplace discrimination across these contexts. Similarly, when recruiting, we did not pay attention to fathers' seniority in the workplace and could not draw conclusions about obstacles to leave-taking across the workplace hierarchy.

These limitations lead to possibilities of further research. To effectively counter fathers' workplace discrimination, their workplaces obstacles and the link to different policies aimed at facilitating fathers' leave-taking require more attention, in Poland and Slovakia, but also elsewhere. In Poland and Slovakia, more research should be undertaken to systematically investigate the variety and prevalence of workplace obstacles than continue constraining fathers' leave-taking after the introduction of different kinds of leave policies for fathers. A particular focus should be dedicated to obstacles across different sectors, employer size and type of position.

## Data Availability

The raw data supporting the conclusions of this article will be made available by the authors, without undue reservation.

## Footnotes

1 A dedicated 28 weeks of paternity leave was introduced in Slovakia in 2022, to be used by fathers directly after the birth of a child and so overlapping with mothers' maternity leave. However, fathers can only draw on the high associated benefits for only two of those weeks, if the mother is on maternity leave and making use of her maternity benefits at the same time. Consequently, if parents want to make use of more than two weeks of fathers' well-paid leave, fathers still they to use the “maternity leave for fathers”.

2 From 2023, following an EU directive, parental leave was extended to 41 weeks, of which 9 weeks are reserved for each parent (Kurowska et al., 2023).
